# Comparative study on heat transfer and friction drag in the flow of various hybrid nanofluids effected by aligned magnetic field and nonlinear radiation

**DOI:** 10.1038/s41598-021-81581-1

**Published:** 2021-02-11

**Authors:** M. Riaz Khan, Mingxia Li, Shipeng Mao, Rashid Ali, Suliman Khan

**Affiliations:** 1 LSEC and ICMSEC, Academy of Mathematics and Systems Science, Chinese Academy of Sciences, School of Mathematical Sciences, University of Chinese Academy of Sciences, Beijing, 100190 People’s Republic of China; 2 School of Science, China University of Geosciences (Beijing), Xueyuan Lu 29, Beijing, 100083 People’s Republic of China; 3 School of Mathematics and Statistics, Central South University, Changsha, 410083 People’s Republic of China

**Keywords:** Computational science, Mechanical engineering, Fluid dynamics

## Abstract

The key purpose of the existing article is to discuss the effects of various hybrid nanofluids and a simple nanofluid over the heat transfer and friction drags along a stretched surface. The various kinds of hybrid nanofluids and a simple nanofluid together with the effects of aligned magnetic field, nonlinear radiation and suction have been taken into consideration. These hybrid nanofluids are prepared by suspending a couple of distinct nanoparticles $Cu$ and $A{l}_{2}{O}_{3}$ into the base fluids ${H}_{2}O$ and ${C}_{2}{H}_{6}{O}_{2}$. The comparison of various graphical results of skin friction coefficient, rate of heat transfer, velocity and temperature for two different hybrid nanofluids $Cu{-}A{l}_{2}{O}_{3}$/${H}_{2}O$, $Cu{-}A{l}_{2}{O}_{3}$/${H}_{2}O{-}{C}_{2}{H}_{6}{O}_{2}$ and a simple nanofluid $A{l}_{2}{O}_{3}$/${H}_{2}O$ is considered. Moreover, the impact of surface stretching, aligned magnetic field and thermal radiation over the velocity, temperature, skin friction coefficient and local Nusselt number are also considered. The outcomes drawn from this modern research is that the hybrid nanofluid $Cu{-}A{l}_{2}{O}_{3}$/${H}_{2}O{-}{C}_{2}{H}_{6}{O}_{2}$ is quite effective in cooling and heating in comparison to the other hybrid nanofluids $Cu{-}A{l}_{2}{O}_{3}$/${H}_{2}O$, $Cu{-}A{l}_{2}{O}_{3}$/${C}_{2}{H}_{6}{O}_{2}$ and a simple nanofluid $A{l}_{2}{O}_{3}/{H}_{2}O$. Based on these findings we could say that the suspension of multiple particles in the composition of two or more base fluids provides a better rate of heat transfer and limits the friction drag.

## Introduction

The characteristics of boundary layer flow across a stretching surface is considerable since it appears in multiple engineering science processing, such as glass blowing, materials produced via extrusion, annealing and tinning of copper wires, fiber spinning, continuous cooling and many others. In the process of manufacturing these sheets, the melt issues from a slit and is subsequently stretched to obtain the appropriate thickness. In such processes, the resulting materials (product) of the required quality depends upon the cooling rate and the stretching process. In the light of these applications and uses, Sakiadis^1^ in 1961 examined the behavior of viscous boundary layer flow over a moving solid surface and after that in 1970, Crane^2^ investigated the flow across a stretching surface. Gupta and Gupta^3^ discussed the flow of heat and mass transfer moving across a stretching surface subject to the mass suction or blowing. The MHD flow of a power-law fluid past a stretching surface was studied by Andersson et al.^4^. Recently Khan et al.^5^ numerically assessed the oblique stagnation point flow of a nanofluid moving over a curved stretching/shrinking sheet. Reddy et al.^6^ evaluated the properties of heat and mass transfer in three-dimensional MHD flow past a stretching sheet occupied by a water-based alumina nanofluid.

The heating and thermal motion of fluid molecules makes a significant contribution in the model of continuum mechanics. In exchange processes the molecules roam out of particles of fluids and are retrieved with molecules drifting in. These exchange processes direct the well-known characteristics of fluids marked as transport properties. In this regard, viscosity is the cause of inward resistance in the momentum transport although in temperature and concentration equations the inner source of heat and mass distribution is the thermal conductivity and diffusion respectively. In the field of dynamics, researchers work out towards the friction drag and heat transfer where they come out to the conclusion that liquids possess smaller thermal conductivity however for solids, they calculated greater thermal conductivity. Reddy et al.^7^ studied the boundary-layer flow of heat and mass transfer over a vertical cone through porous media filled with a Cu–water and Ag–water nanofluid. The comparative study of $A{l}_{2}{O}_{3}$ and $Ti{O}_{2}$ nanofluid flow over a wedge with non-linear thermal radiation was discussed by Sreedevi et al.^8^. Prabhavathi et al.^9^ elaborated the boundary layer MHD heat and mass transfer flow over a vertical cone embedded in porous media filled with $A{l}_{2}{O}_{3}$–water and Cu–water nanofluid. Some other investigations towards the effect of single and multiple nanoparticles on the thermal conductivity, and heat transfer enhancement is given in^10–19^. The higher thermal conductivity improves the rate of heat transfer. Therefore, the suspension of solid nanoparticles in the base liquids causes to enhance the rate of heat transfer in the liquids. Ultimately, to gain the remarkable refinement in the thermal conductivity, an alternative class of fluids titled as nanofluids are introduced. The nanofluid is a colloidal solution consisting of nano-scale solid particles. Such type of fluids spotted wonderful improvement in useful applications and software’s containing microelectronics, cooling and refrigeration, processors of versatile PCs, solar thermal, transportation and similarly in high performance military correspondence equipment’s, etc. Even though nanofluids succor the passion of researchers and engineers in the thermal efficiency, yet a supreme type of fluid is in search being presently. In respondent to these, a superior quality of nanofluids namely hybrid nanofluids have been introduced which are holding strong thermal conductivity instead of nanofluids. Thus, the ongoing effort particularly dealing with hybrid nanofluids which is the key purpose of the authors intended to improve the transfer of heat.

Hybrid nanofluid is a modern family of nanofluid which are produced by suspending distinct multiple nanoparticles in the base fluids while primarily addressed by Jana et al.^20^. The accumulation of small fraction of metal nanotubes or nanoparticles within the nanoparticles of an oxide or metal which is already existing in a base liquid may surprisingly boost the thermal features. The benefits of hybrid nanofluids are the extensive advance thermal conductivity, stability, rectified heat transfer, positive effects of individual suspension, and combined influence of nanomaterials. The areas of implementation of hybrid nanofluids differs widely in almost all the areas of heat transfer, for instance, generator cooling, coolant in machining, thermal capacity, electronic cooling, heating and cooling in houses, vehicle thermal management or motor cooling, transformer cooling, atomic framework cooling, refrigeration, medication reduction, welding, defense, heat pipe, biomedical, boats and space airplanes with more effective efficiency higher than that of nanofluids applications. These valuable qualities fascinated the attention of researchers to operate towards the hybrid nanofluid in the daily life problems of heat transfer. Various introductory analysis has commenced remarkable improvement by applying these kinds of techniques of hybrid nanofluids^21–24^.

The on-going efforts is carried out in accordance with the two-phase model bearing in mind the notion of heat transfer in liquids and solids. Two different kinds of hybrid nanofluids viz, $Cu{-}A{l}_{2}{O}_{3}$/${H}_{2}O{-}{C}_{2}{H}_{6}{O}_{2}$, $Cu{-}A{l}_{2}{O}_{3}$/${H}_{2}O$, and a simple nanofluid $A{l}_{2}{O}_{3}/{H}_{2}O$ are taken into consideration by suspending the distinct multiple nanoparticles $(Cu$ and $A{l}_{2}{O}_{3}$) in the base fluids (${H}_{2}O$ and ${C}_{2}{H}_{6}{O}_{2}$). In the former analysis, the flow of the hybrid nanofluid was just confined to one base fluid and two nanoparticles. However, few papers study the flow of a hybrid nanofluid with two different nanoparticles and two different base fluids. This motivates us to check the heat transfer and friction drag under the effect of these novel kind of hybrid nanofluids in viscous flow model. The effects of nonlinear thermal radiation, inclined magnetic field and mass suction are also considered. To the best knowledge of the authors no one in the past studied such type of flow problem. Thus, the present study completely diverges from the earlier one. The key objective of our study was to check only that how much these new kinds of fluid comparatively affects the flow properties. Since the existence of large number of nanoparticles in various base fluid improves the density of the associated hybrid nanofluids and thus the temperature of the coupled nanoparticles in the base fluids may be affected. Thus, hybrid nanofluids make superior performance in electronic components and other appliances. The current investigation is further useful in refrigerators for stabilizing their rate of cooling. This modern type of work will allure countless other researchers due to their unlimited wonderful latest applications which stimulated us to assess the current effort.

## Basic equations

Consider the steady two-dimensional viscous flow of various hybrid nanofluids and a simple nanofluid along a stretched surface. In this flow, the effects of nonlinear thermal radiation, inclined magnetic field and mass suction are also considered. Two different nanoparticles $Cu$ and $A{l}_{2}{O}_{3}$ are synthesized with two different base fluids ${H}_{2}O$ and ${C}_{2}{H}_{6}{O}_{2}$. Initially, the nanoparticles $Cu$ and $A{l}_{2}{O}_{3}$ are individually suspended in the base fluid ${H}_{2}O$ and ${C}_{2}{H}_{6}{O}_{2}$ to prepare the hybrid nanofluid $Cu{-}A{l}_{2}{O}_{3}$/${H}_{2}O$ and a simple nanofluid $A{l}_{2}{O}_{3}$/${H}_{2}O$. Secondly the same nanoparticles are combined with the two base fluids ${H}_{2}O$ and ${C}_{2}{H}_{6}{O}_{2}$ to produce the hybrid nanofluid $Cu{-}A{l}_{2}{O}_{3}$/${H}_{2}O{-}{C}_{2}{H}_{6}{O}_{2}$. Further, the existence of an aligned magnetic field with an acute angle $\beta$ are assumed. The thermophysical properties and their mathematical expressions for above nanoparticles and base fluids are individually specified in Tables 1 and 2, whereas the governing flow equations and their corresponding boundary conditions are stated in Eqs. (1–4)^25,26^

**Table 1:** Thermophysical properties of base fluids and nanoparticles^27,28^.

Thermophysical properties	Water ($${H}_{2}O):$$($${f}_{1})$$	Ethylene glycol ($${C}_{2}{H}_{6}{O}_{2}$$): ($${f}_{2})$$	Cupper (C $$u)$$ ($${s}_{1})$$	Alumina ($$A{l}_{2}{O}_{3}$$):($${s}_{2})$$
${C}_{p}$ (J/kgK)	4179	2430	385	765
$\rho$ (kg/m^3^)	997.1	1115	8933	3970
$k$ (W/mK)	0.613	0.0803	401	40
$\sigma$ (S/m)	${5.5\times 10}^{-6}$	${1\times 10}^{-7}$	${59.6\times 10}^{6}$	${35\times 10}^{6}$

**Table 2:** Mathematical expressions for the thermophysical properties of ${Al}_{2}{O}_{3}{-}{H}_{2}O$, $Cu{-}A{l}_{2}{O}_{3}/{H}_{2}O$ or $Cu{-}A{l}_{2}{O}_{3}/{C}_{2}{H}_{6}{O}_{2}$ and $Cu{-}A{l}_{2}{O}_{3}/{H}_{2}O{-}{C}_{2}{H}_{6}{O}_{2}$^27^.

Properties	Nanofluid (${Al}_{2}{O}_{3}{-}{H}_{2}O$)
Density	${\rho }_{nf}=\varphi {\rho }_{s}+\left(1-\varphi \right){\rho }_{f}$
Heat capacity	${\left(\rho {C}_{p}\right)}_{nf}=\varphi {\left(\rho {C}_{p}\right)}_{s}+\left(1-\varphi \right){\left(\rho {C}_{p}\right)}_{f}$
Electrical conductivity	${\sigma }_{nf}=\left(1-\varphi \right){\sigma }_{f}+\varphi {\sigma }_{s}$
Viscosity	${\mu }_{nf}=\frac{{\mu }_{f}}{{\left(1-\varphi \right)}^{2.5}}$
Thermal diffusivity	${\alpha }_{nf}=\frac{{k}_{nf}}{{\left(\rho {C}_{p}\right)}_{nf}}$
Thermal conductivity	$\frac{{k}_{nf}}{{k}_{f}}=\frac{\left(\frac{{k}_{s}}{{k}_{f}}+2\right)-2\varphi \left(1-\frac{{k}_{s}}{{k}_{f}}\right)}{\left(\frac{{k}_{s}}{{k}_{f}}+2\right)+\varphi \left(1-\frac{{k}_{s}}{{k}_{f}}\right)}$
**Hybrid nanofluid (**${\varvec{C}}{\varvec{u}}{-}{\varvec{A}}{{\varvec{l}}}_{2}{{\varvec{O}}}_{3}/{{\varvec{H}}}_{2}{\varvec{O}})$** or **$({\varvec{C}}{\varvec{u}}{-}{\varvec{A}}{{\varvec{l}}}_{2}{{\varvec{O}}}_{3}/{{\varvec{C}}}_{2}{{\varvec{H}}}_{6}{{\varvec{O}}}_{2}$**)**	
Density	${\rho }_{hnf}=\left[\left\{\left(1-{\varphi }_{1}\right){\rho }_{f}+{\varphi }_{1}{\rho }_{{s}_{1}}\right\}\left(1-{\varphi }_{2}\right)\right]+{\varphi }_{2}{\rho }_{{s}_{2}}$
Heat capacity	${\left(\rho {C}_{p}\right)}_{hnf}=\left[\left\{\left(1-{\varphi }_{1}\right){\left(\rho {C}_{p}\right)}_{f}+{\varphi }_{1}{\left(\rho {C}_{p}\right)}_{{s}_{1}}\right\}\left(1-{\varphi }_{2}\right)\right]+{\varphi }_{2}{\left(\rho {C}_{p}\right)}_{{s}_{2}}$
Viscosity	${\mu }_{hnf}=\frac{{\mu }_{f}}{{\left(1-{\varphi }_{1}\right)}^{2.5}{\left(1-{\varphi }_{2}\right)}^{2.5}}$
Thermal conductivity	$\frac{{k}_{hnf}}{{k}_{bf}}=\frac{{k}_{{s}_{2}}+\left(n-1\right){k}_{bf}-\left(n-1\right){\varphi }_{2}\left({k}_{bf}-{k}_{{s}_{2}}\right)}{{k}_{{s}_{2}}+\left(n-1\right){k}_{bf}+{\varphi }_{2}\left({k}_{bf}-{k}_{{s}_{2}}\right)}$ where $\frac{{k}_{bf}}{{k}_{f}}=\frac{{k}_{{s}_{1}}+\left(n-1\right){k}_{f}-\left(n-1\right){\varphi }_{1}\left({k}_{f}-{k}_{{s}_{1}}\right)}{{k}_{{s}_{1}}+\left(n-1\right){k}_{f}+{\varphi }_{1}\left({k}_{f}-{k}_{{s}_{1}}\right)}$
Electrical conductivity	$\frac{{\sigma }_{hnf}}{{\sigma }_{bf}}=\frac{{\sigma }_{{s}_{2}}+2{\sigma }_{bf}-2{\varphi }_{2}({\sigma }_{bf}-{\sigma }_{{s}_{2}})}{{\sigma }_{{s}_{2}}+2{\sigma }_{bf}+{\varphi }_{2}({\sigma }_{bf}-{\sigma }_{{s}_{2}})}$ where $\frac{{\sigma }_{bf}}{{\sigma }_{f}}=\frac{{\sigma }_{{s}_{1}}+2{\sigma }_{f}-2{\varphi }_{1}({\sigma }_{f}-{\sigma }_{{s}_{1}})}{{\sigma }_{{s}_{1}}+2{\sigma }_{f}+{\varphi }_{1}({\sigma }_{f}-{\sigma }_{{s}_{1}})}$
**Hybrid nanofluid (**${\varvec{C}}{\varvec{u}}{-}{\varvec{A}}{{\varvec{l}}}_{2}{{\varvec{O}}}_{3}/{{\varvec{H}}}_{2}{\varvec{O}}{-}{{\varvec{C}}}_{2}{{\varvec{H}}}_{6}{{\varvec{O}}}_{2}$**)**	
Density	${\rho }_{hnf}=\left[\left\{\left(1-{\varphi }_{1}\right)({\rho }_{f1}+{\rho }_{f2})+{\varphi }_{1}({\rho }_{{s}_{1}}+{\rho }_{{s}_{2}})\right\}\left(1-{\varphi }_{2}\right)\right]+{\varphi }_{2}({\rho }_{{s}_{1}}+{\rho }_{{s}_{2}})$
Heat capacity	${\left(\rho {C}_{p}\right)}_{hnf}=\left[\left(1-{\varphi }_{1}\right)\left\{{\left(\rho {C}_{p}\right)}_{f1}+{\left(\rho {C}_{p}\right)}_{f2}\right\}+{\varphi }_{1}\left\{{\left(\rho {C}_{p}\right)}_{{s}_{1}}+{\left(\rho {C}_{p}\right)}_{{s}_{2}}\right\}\right]\left(1-{\varphi }_{2}\right)+{\varphi }_{2}\left\{{{\left(\rho {C}_{p}\right)}_{{s}_{1}}+\left(\rho {C}_{p}\right)}_{{s}_{2}}\right\}$
Viscosity	${\mu }_{hnf}=\frac{{\mu }_{f1}+{\mu }_{f2}}{{\left(1-{\varphi }_{1}\right)}^{2.5}{\left(1-{\varphi }_{2}\right)}^{2.5}}.$
Thermal conductivity	$\frac{{k}_{hnf}}{{k}_{bf1}+{k}_{bf2}}=\frac{{{(k}_{{s}_{1}}+k}_{{s}_{2}})+\left(n-1\right)({k}_{bf1}+{k}_{bf2})-\left(n-1\right){\varphi }_{2}\left\{\left({k}_{bf1}+{k}_{bf2})-({k}_{{s}_{1}}{+k}_{{s}_{2}}\right)\right\}}{{{(k}_{{s}_{1}}+k}_{{s}_{2}})+\left(n-1\right)\left({k}_{bf1}+{k}_{bf2}\right)+{\varphi }_{2}\left\{\left({k}_{bf1}+{k}_{bf2})-({k}_{{s}_{1}}{+k}_{{s}_{2}}\right)\right\}} .$ where $\frac{{k}_{bf1}+{k}_{bf2}}{{k}_{f1+{k}_{f2}}}=\frac{{(k}_{{s}_{1}}+{k}_{{s}_{2}})+\left(n-1\right)\left({k}_{f1}+{k}_{f2}\right)-\left(n-1\right){\varphi }_{1}\left\{\left({k}_{f1}+{k}_{f2})-({k}_{{s}_{1}}+{k}_{{s}_{2}}\right)\right\}}{{(k}_{{s}_{1}}+{k}_{{s}_{2}})+\left(n-1\right)\left({k}_{f1}+{k}_{f2}\right)+{\varphi }_{1}\left\{\left({k}_{f1}+{k}_{f2})-({k}_{{s}_{1}}+{k}_{{s}_{2}}\right)\right\}} .$
Electrical conductivity	$\frac{{\sigma }_{hnf}}{{\sigma }_{{bf}_{1}}+{\sigma }_{{bf}_{2}}}=\frac{{(\sigma }_{{s}_{1}}+{\sigma }_{{s}_{2}})+2{(\sigma }_{{bf}_{1}}+{\sigma }_{{bf}_{2}})-2{\varphi }_{2}\left\{({\sigma }_{{bf}_{1}}+{\sigma }_{{bf}_{2}})-{(\sigma }_{{s}_{1}}+{\sigma }_{{s}_{2}})\right\}}{{(\sigma }_{{s}_{1}}+{\sigma }_{{s}_{2}})+2{(\sigma }_{{bf}_{1}}+{\sigma }_{{bf}_{2}})+{\varphi }_{2}\left\{({\sigma }_{{bf}_{1}}+{\sigma }_{{bf}_{2}})-{(\sigma }_{{s}_{1}}+{\sigma }_{{s}_{2}})\right\}}$ where $\frac{{\sigma }_{{bf}_{1}}+{\sigma }_{{bf}_{2}}}{{\sigma }_{{f}_{1}}+{\sigma }_{{f}_{2}}}=\frac{{(\sigma }_{{s}_{1}}+{\sigma }_{{s}_{2}})+2({\sigma }_{{f}_{1}}+{\sigma }_{{f}_{2}})-2{\varphi }_{1}\left\{({\sigma }_{{f}_{1}}+{\sigma }_{{f}_{2}})-{(\sigma }_{{s}_{1}}+{\sigma }_{{s}_{2}})\right\}}{{(\sigma }_{{s}_{1}}+{\sigma }_{{s}_{2}})+2\left({\sigma }_{{f}_{1}}+{\sigma }_{{f}_{2}}\right)+{\varphi }_{1}\left\{({\sigma }_{{f}_{1}}+{\sigma }_{{f}_{2}})-{(\sigma }_{{s}_{1}}+{\sigma }_{{s}_{2}})\right\}}$

1 $$\frac{\partial u}{\partial x}+\frac{\partial u}{\partial y}=0,$$

2 $$u\frac{\partial u}{\partial x}+v\frac{\partial u}{\partial y}=\frac{{\mu }_{hnf}}{{\rho }_{hnf}}\frac{{\partial }^{2}u}{\partial {y}^{2}}-\frac{{\sigma }_{hnf}{{B}_{0}}^{2}}{{\rho }_{hnf}}{\mathrm{sin}}^{2}\beta u,$$

3 $${\left({\rho C}_{p}\right)}_{hnf}\left(u\frac{\partial T}{\partial x}+v\frac{\partial T}{\partial y}\right)={k}_{hnf}\frac{{\partial }^{2}T}{\partial {y}^{2}}-\frac{\partial {q}_{r}}{\partial y}.$$

The boundary constraints assigned for the flow equations are

4 $$\left. {\begin{array}{*{20}r} \hfill {u = u\left( x \right),\;v = v\left( x \right),\;T = T_{w} \;at\;y = 0} \\ \hfill {u = u_{e} \left( x \right),\;T = T_{\infty } \;as\;y \to \infty } \\ \end{array} } \right\}$$

where $u\left(x\right)=Gx$, $v\left(x\right)={-\upsilon }_{x},$ ${u}_{e}\left(x\right)=0,$ and $G$ is a nondimensional constant which represent the stretching of sheet. $T$ is the temperature of the nanofluid, ${T}_{\infty }$ is the temperature of the nanofluid far away from the wall and ${q}_{r}$ is the radiative heat flux. The Rosseland’s relation for radiative heat flux ${q}_{r}$ is given by^26, 29^

5 $${q}_{r}=\frac{-4{\sigma }^{*}}{3{k}^{*}}\frac{\partial {T}^{4}}{\partial y}$$

In the above equation, ${k}^{*}$ interpret the mean absorption coefficient and ${\sigma }^{*}$ marks the Stefan-Boltzmann constant.

Applying the Taylor series expanding for simplifying the nonlinear temperature term ${T}^{4}$ about ${T}_{\infty }$ and leave out the higher-order terms, we achieve

6 $${T}^{4}\approxeq 4{T}_{\infty }^{3}T-3{\sigma }^{*}{T}_{\infty }^{4}$$

By putting Eq. (6) in Eq. (5), we obtain

7 $${q}_{r}=\frac{-16{\sigma }^{*}{T}_{\infty }^{3}}{3{k}^{*}}\frac{\partial T}{\partial y}$$

Now, Eq. (3) and Eq. (8) yields

8 $${\left({\rho C}_{p}\right)}_{hnf}\left(u\frac{\partial T}{\partial x}+v\frac{\partial T}{\partial y}\right)={k}_{hnf}\frac{{\partial }^{2}T}{\partial {y}^{2}}+\frac{16{\sigma }^{*}{T}_{\infty }^{3}}{3{k}^{*}}\frac{{\partial }^{2}T}{\partial {y}^{2}}$$

The similarity variables are defined as

9 $$u=mx{f}^{^{\prime}}\left(\eta \right), v=-\sqrt{\frac{m{\mu }_{f}}{{\rho }_{f}}}f\left(\eta \right), \eta =\sqrt{\frac{m{\rho }_{f}}{{\mu }_{f}}}y, \theta \left(y\right)=\frac{T-{T}_{\infty }}{{T}_{w}-{T}_{\infty }},$$

where $m$ is a positive constant ie $m>0.$

Using Eq. (9), Eq. (1) is satisfied automatically, whereas Eqs. (2) and (8) reduces to

10 $$\frac{{\mu_{hnf} }}{{\mu_{f} }}\frac{{\rho_{f} }}{{\rho_{hnf} }}f^{\prime\prime\prime} - \left( {f^{\prime}} \right)^{2} + ff^{\prime\prime} - M^{2} \frac{{\sigma_{hnf} }}{{\sigma_{f} }}\frac{{\rho_{f} }}{{\rho_{hnf} }}\sin^{2} \beta f^{\prime} = 0 ,$$

11 $$\frac{1}{Pr}\frac{{k_{hnf} }}{{k_{f} }}\left( {1 + \frac{4}{3}Rd} \right)\theta^{\prime\prime} + \frac{{\left( {\rho C_{p} } \right)_{hnf} }}{{\left( {\rho C_{p} } \right)_{f} }}f\theta^{\prime} = 0.$$

12 $$\left. {\begin{array}{*{20}r} \hfill {f\left( 0 \right) = S,\;f^{\prime}\left( 0 \right) = \gamma ,\;\theta \left( 0 \right) = 1,} \\ \hfill {f\left( \eta \right) = 0,\;\theta \left( \eta \right) = 0\;as\;\eta \to \infty } \\ \end{array} } \right\}$$

where $S=\frac{{\upsilon }_{w}}{\sqrt{a{\upsilon }_{f}}}$ represents suction parameter, $\gamma =\frac{G}{m}$ represents the stretching parameter, $Pr=\frac{{\mu }_{f}}{{\rho }_{f}}\frac{{\left(\rho {C}_{p}\right)}_{f}}{{k}_{f}}$ is a Prandtl number, $Rd=\frac{4{\sigma }^{*}{T}_{\infty }^{3}}{{k}_{hnf}{k}^{*}}$ defines the radiation parameter and ${M}^{2}=\frac{{\sigma }_{f}{{B}_{0}}^{2}}{a{\rho }_{f}}$ identifies the Hartmann number.

The friction drags (skin friction coefficient) and heat transfer rate (local Nusselt number) over the stretched surface are determined as

13 $${C}_{f}=\frac{{\tau }_{w}}{\frac{1}{2}{\rho }_{f}{U}_{w}^{2}}, Nu=\frac{x{q}_{w}}{{k}_{f}\left({T}_{w}-{T}_{\infty }\right)}$$

where ${\tau }_{w}$ constitute local wall shear stress and ${q}_{w}$ is the local heat flux and are determined as

14 $${{\tau }_{w}=\left.{\mu }_{hnf}\frac{\partial u}{\partial y}\right|}_{y=0} \mathrm{and} {q}_{w}=-{k}_{hnf}{\left.\frac{\partial T}{\partial y}\right|}_{y=0} .$$

Taking advantage of similarity variables defined in Eq. (9), the combination of system (13) and (14) provides system (15)

15 $$\left. {\begin{array}{*{20}c} {\frac{1}{2}\left( {Re_{x} } \right)^{{{\raise0.7ex\hbox{$1$} \!\mathord{\left/ {\vphantom {1 2}}\right.\kern-\nulldelimiterspace} \!\lower0.7ex\hbox{$2$}}}} C_{f} = \frac{{\mu_{hnf} }}{{\mu_{f} }}f^{\prime\prime}\left( 0 \right) ,} \\ {\left( {Re_{x} } \right)^{{ - \frac{1}{2}}} Nu = - \frac{{k_{hnf} }}{{k_{f} }}\left( {1 + \frac{4}{3}Rd} \right)\theta^{\prime}\left( 0 \right).} \\ \end{array} } \right\}.$$

In (15), ${Re}_{x}=\frac{{\rho }_{f}{U}_{w}^{2}}{{a\mu }_{f}}$ represents the local Reynolds number.

## Results and discussion

**Figure 1:**
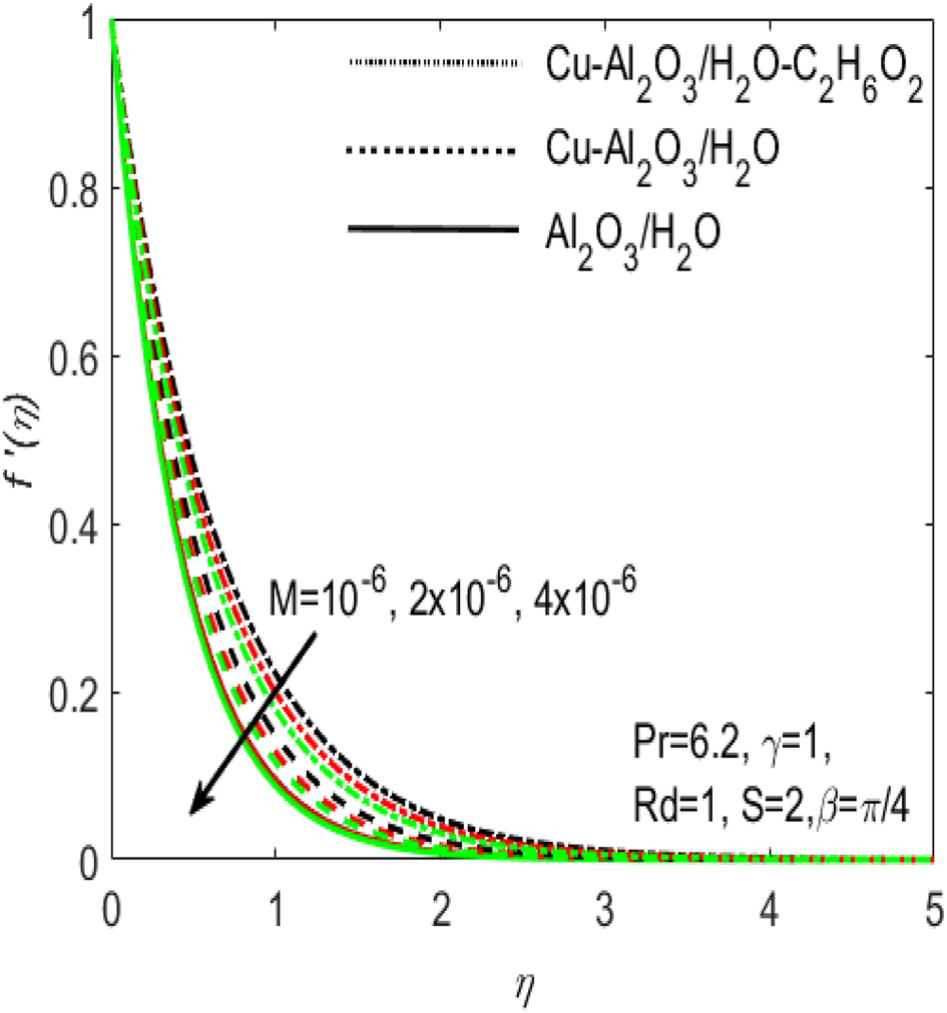
Influence of $M$ on ${f}^{^{\prime}}\left(\eta \right)$ for different hybrid nanofluids and simple nanofluid.

**Figure 2:**
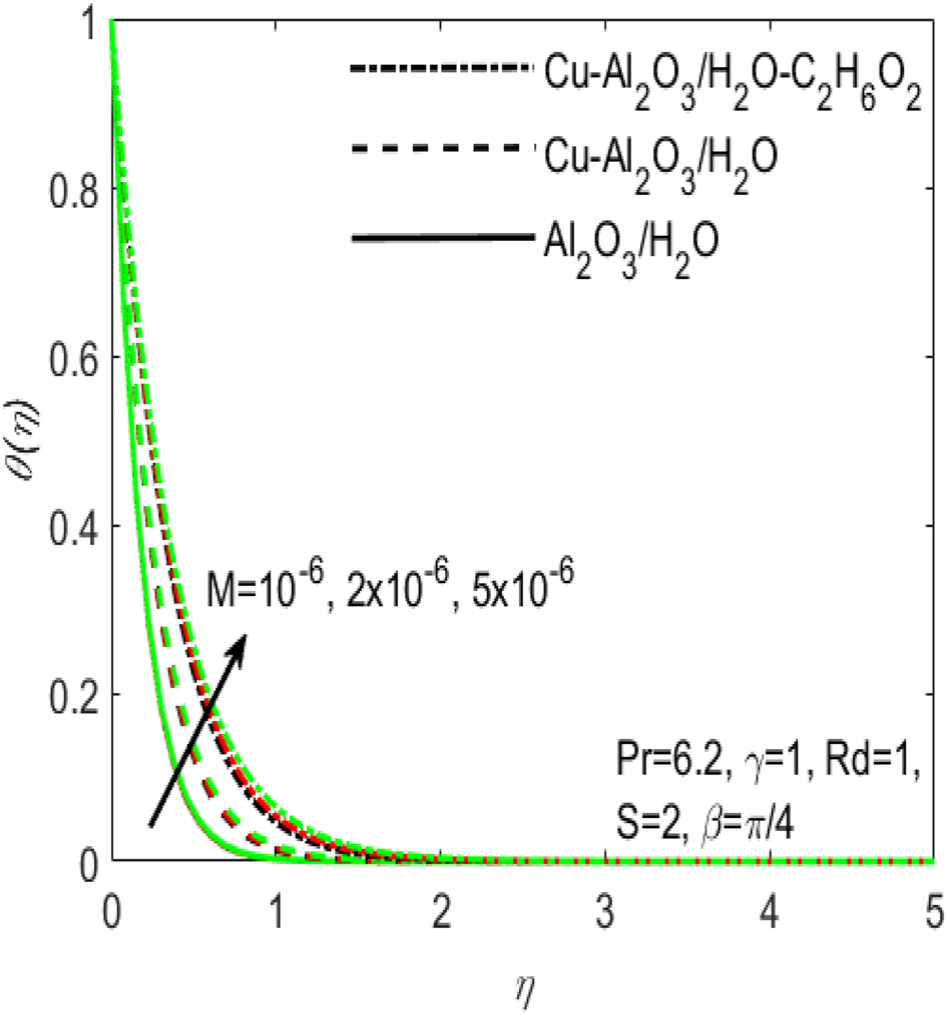
Influence of $M$ on $\theta \left(\eta \right)$ for different hybrid fluids and simple nanofluid.

**Figure 3:**
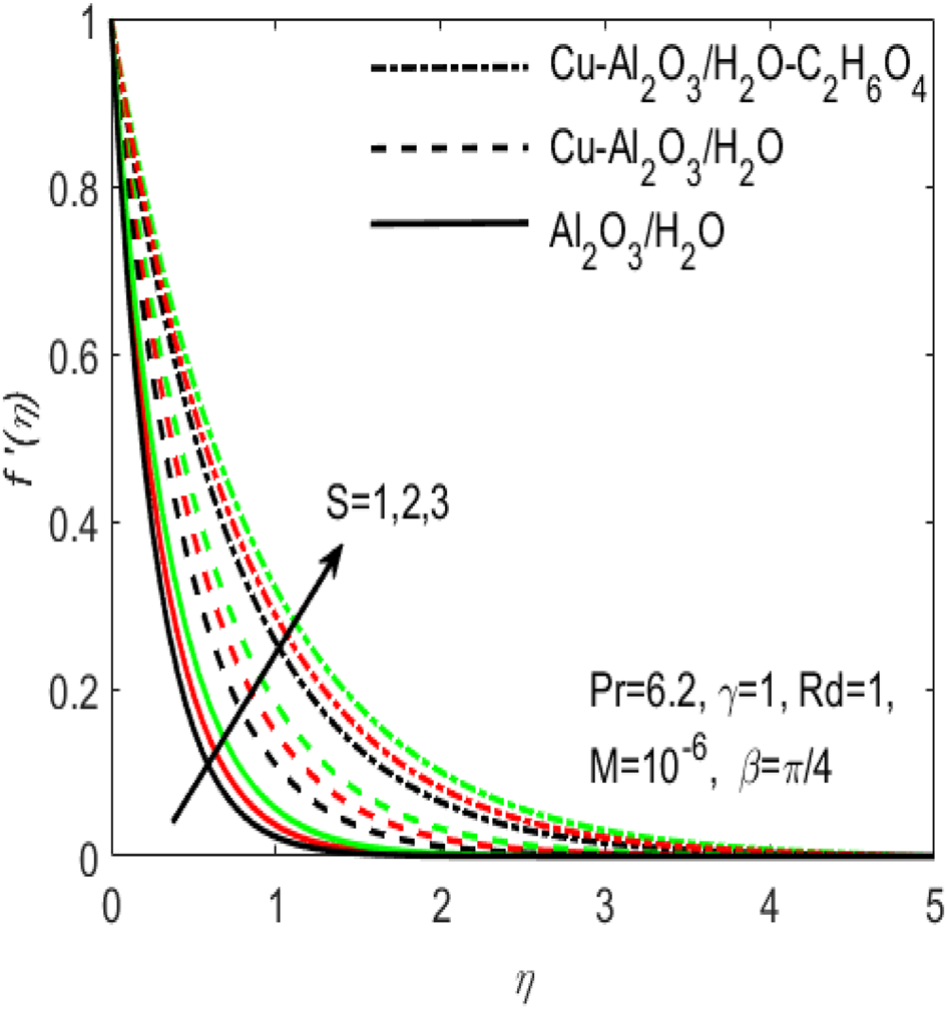
Influence of $S$ on ${f}^{^{\prime}}\left(\eta \right)$ for different hybrid nanofluids and simple nanofluid.

**Figure 4:**
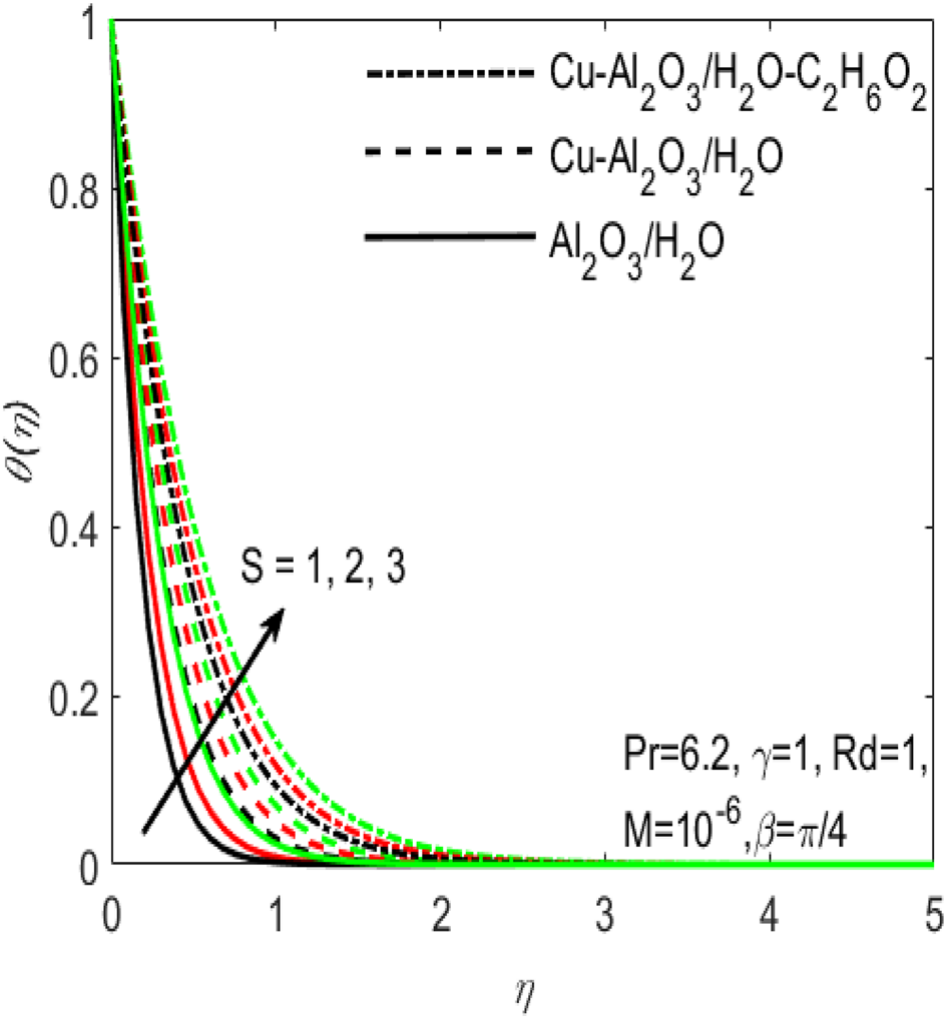
Influence of $S$ on $\theta \left(\eta \right)$ for different hybrid nanofluids and simple nanofluid.

**Figure 5:**
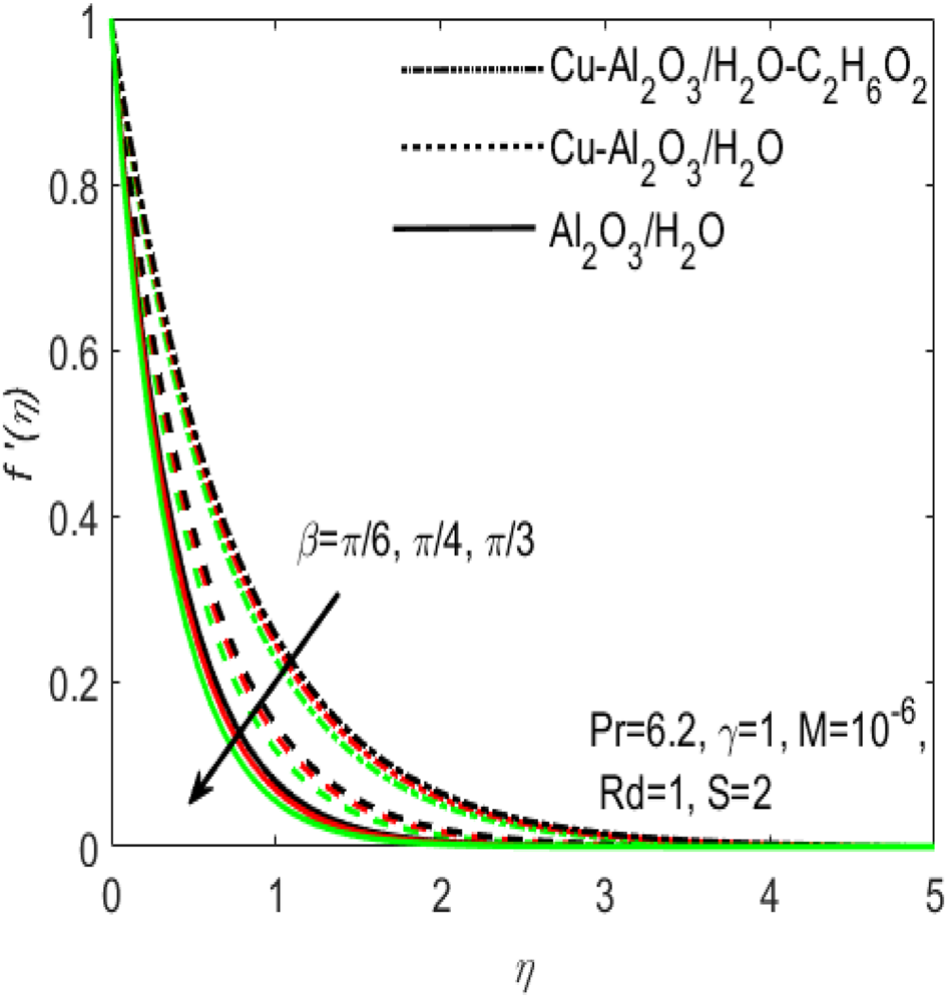
Influence of $\beta$ on ${f}^{^{\prime}}\left(\eta \right)$ for different hybrid nanofluids and simple nanofluid.

**Figure 6:**
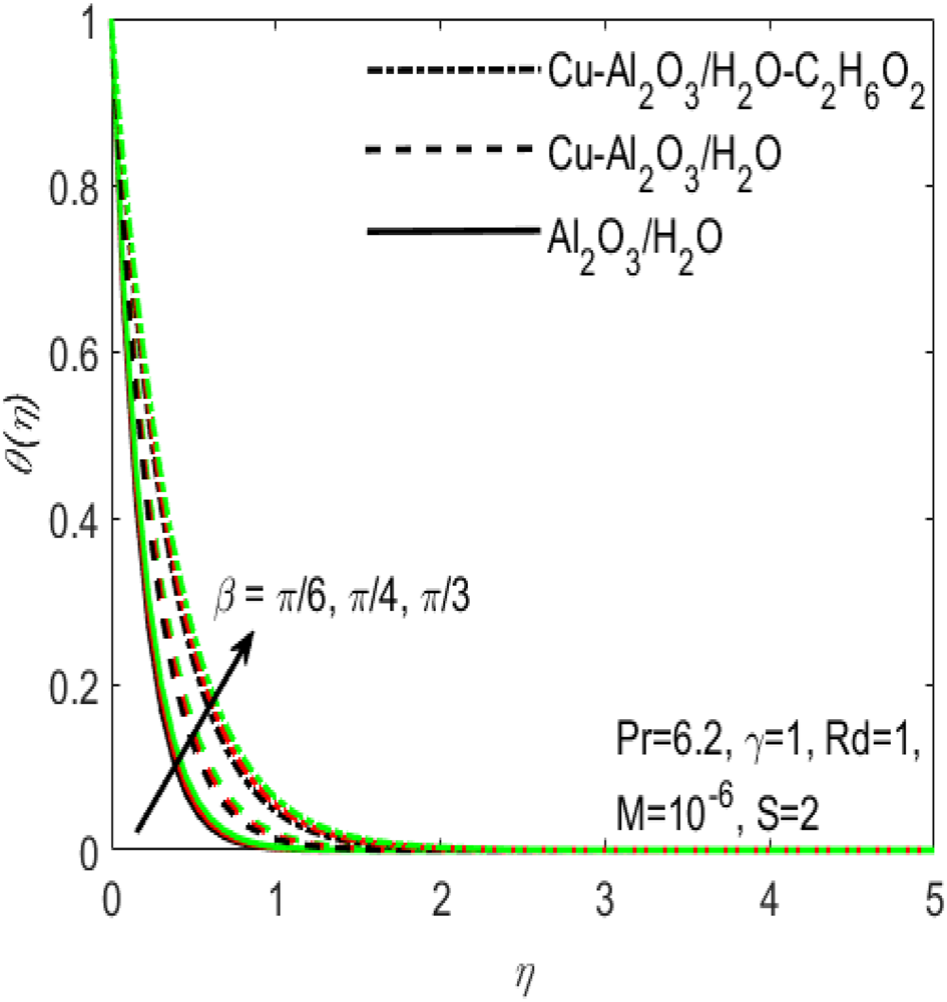
Influence of $\beta$ on $\theta \left(\eta \right)$ for different hybrid nanofluids and simple nanofluid.

**Figure 7:**
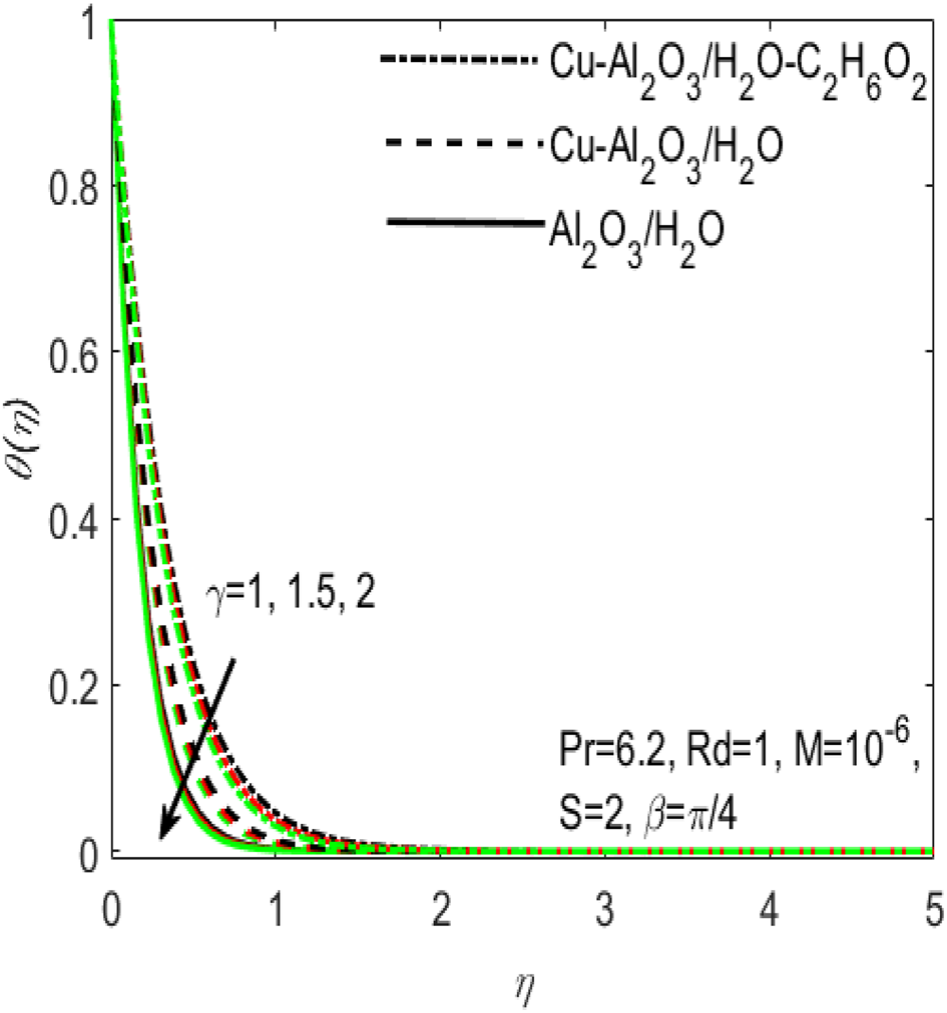
Influence of $\gamma$ on $\theta \left(\eta \right)$ for different hybrid nanofluids and simple nanofluid.

**Figure 8:**
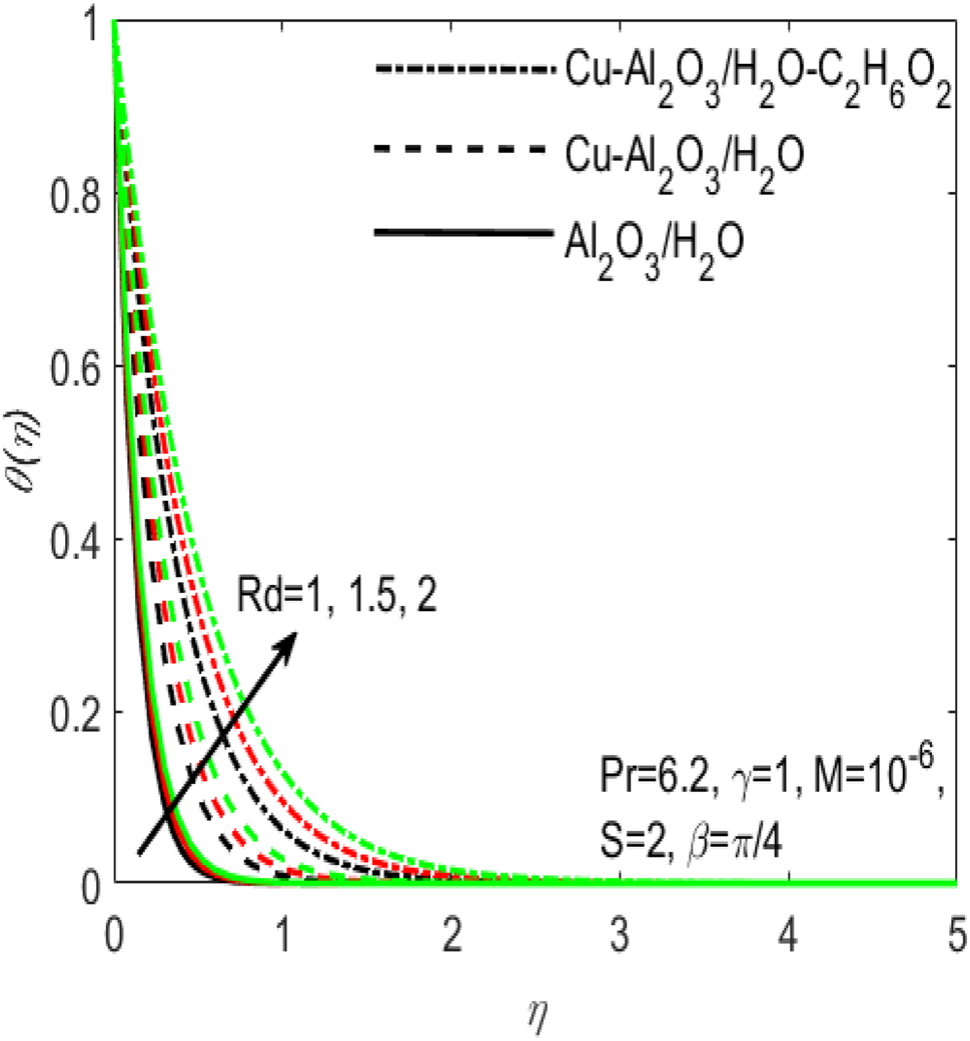
Influence of $Rd$ on $\theta \left(\eta \right)$ for different hybrid nanofluids and simple nanofluid.

**Figure 9:**
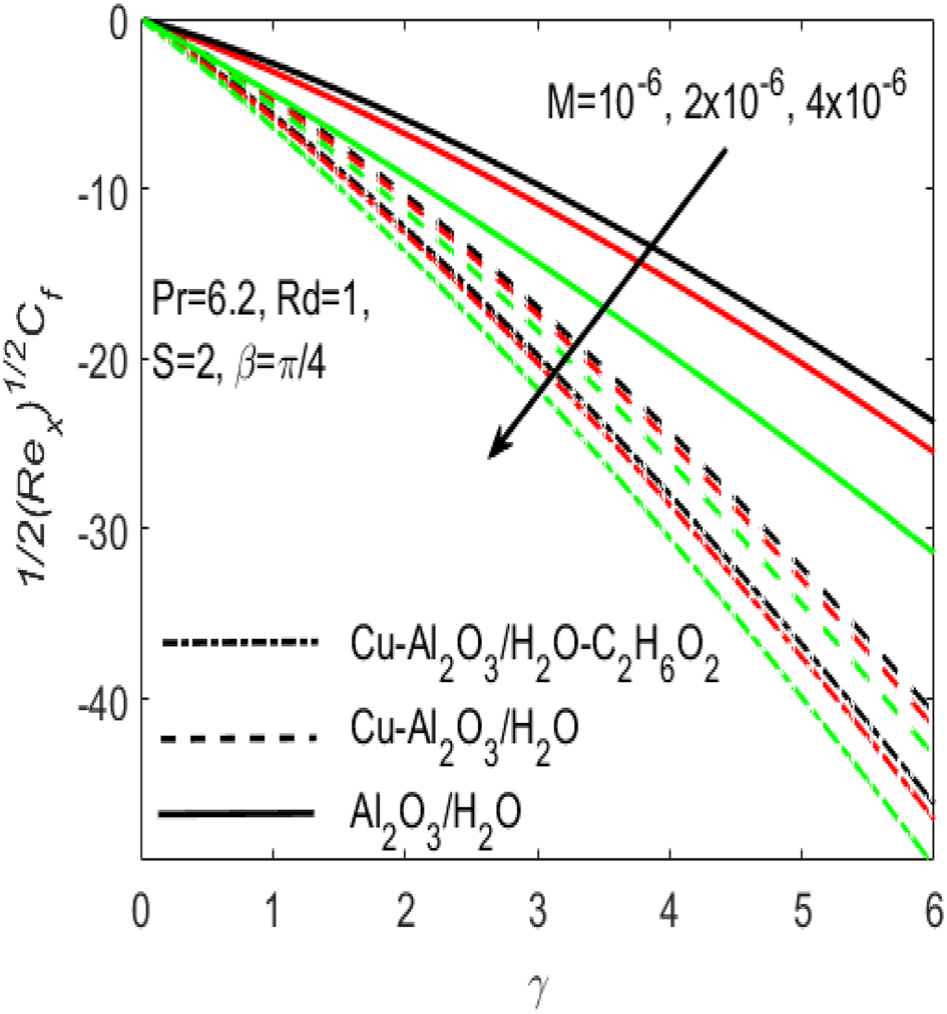
Influence of $M$ on ${C}_{f}$ for different hybrid nanofluids and simple nanofluid.

**Figure 10:**
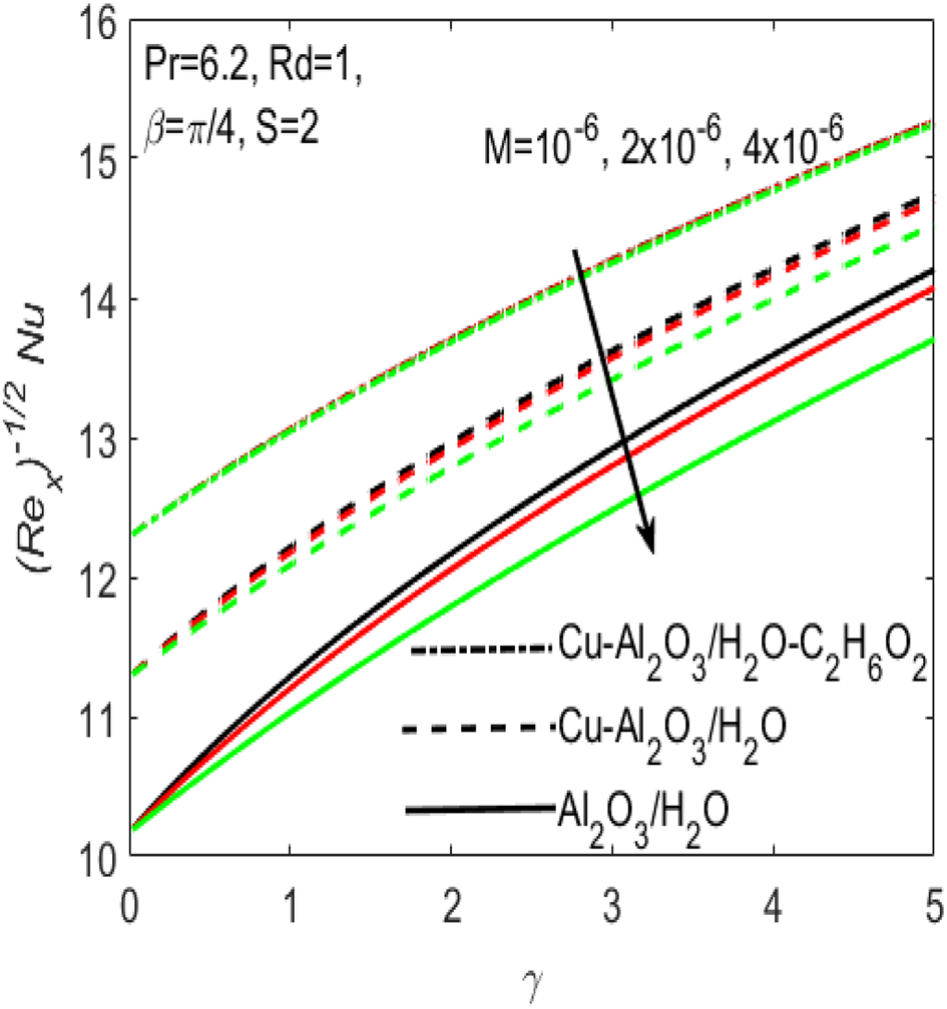
Influence of $M$ on $Nu$ for different hybrid nanofluids and simple nanofluid.

**Figure 11:**
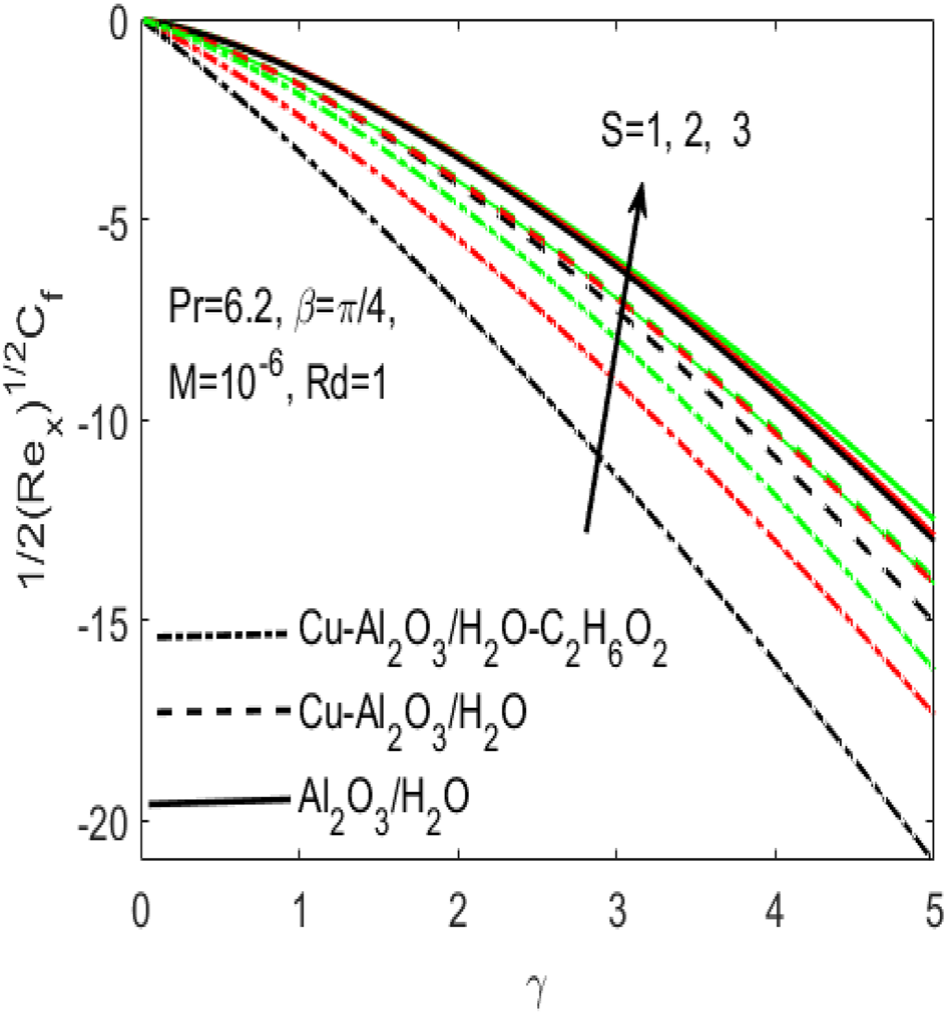
Influence of $S$ on ${C}_{f}$ for different hybrid nanofluids and simple nanofluid.

**Figure 12:**
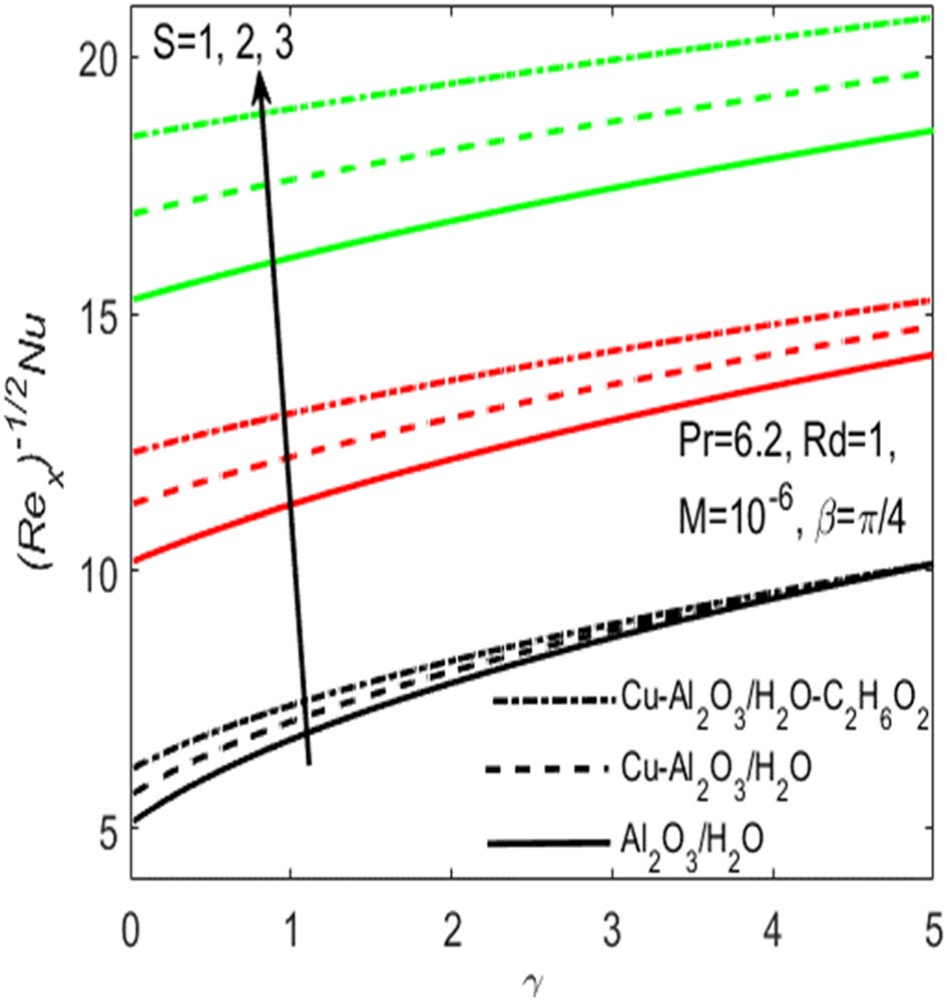
Influence of $S$ on $Nu$ for different hybrid nanofluids and simple nanofluid.

**Figure 13:**
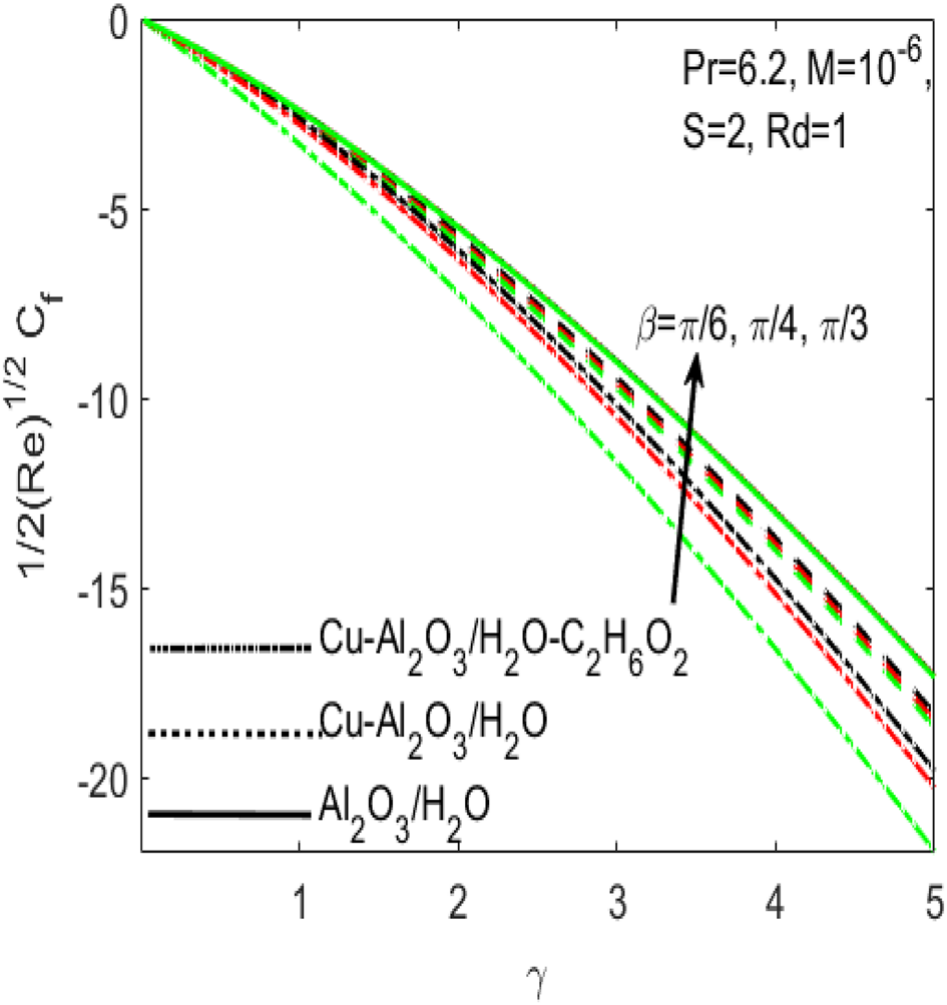
Influence of $\beta$ on ${C}_{f}$ for different hybrid nanofluids and simple nanofluid.

**Figure 14:**
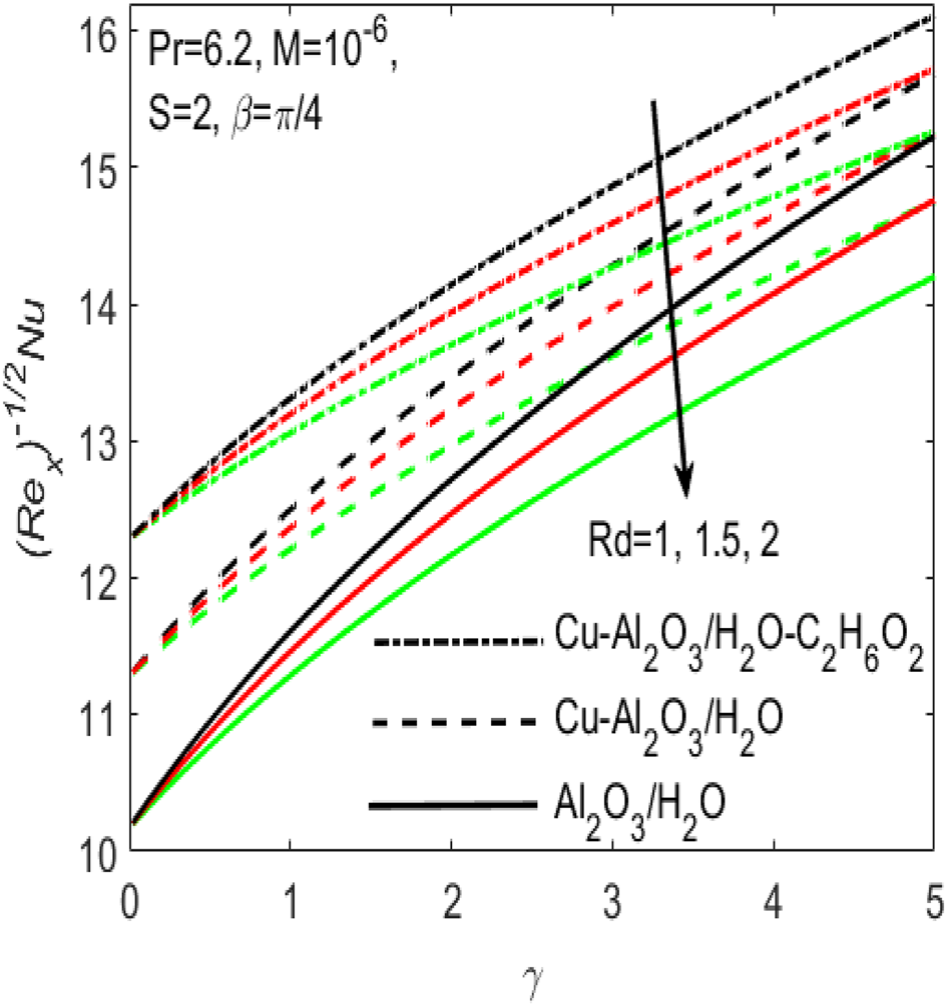
Influence of $\gamma$ on $Nu$ for different hybrid nanofluids and simple nanofluid.

In this section, we have conducted the numerical solutions of nonlinear and non-dimensional Eqs. (10) and (11) coupled with the boundary conditions (12) for several hybrid nanofluids consisting of different nanoparticles like cupper, $(\mathrm{Cu})$ and alumina $({\mathrm{Al}}_{2}{\mathrm{O}}_{3})$ and the base fluids water ${(\mathrm{H}}_{2}\mathrm{O})$ and Ethylene glycol ${(\mathrm{C}}_{2}{\mathrm{H}}_{6}{\mathrm{F}}_{2})$. The different outcomes are found by providing the MATLAB function bvp4c and are displayed graphically in Figs. 1, 2, 3, 4, 5, 6, 7, 8, 9, 10, 11, 12, 13, and 14. In order to enable the use of bvp4c package, it is necessary to reduce our problem into a system of first-order ordinary differential equations. The boundary value problem solver bvp4c requires three pieces of information: the equation to be solved, its associated boundary conditions, and the initial guess for the solution. Bvp4c is basically a finite difference procedure that performs the three stage Lobatto IIIa formula, during which the first-class continuous solution is granted by means of the collocation formula with the accuracy of fourth order. The selection of mesh and error control are based on the residual of the continuous solution. A comparison of the present numerical results of *f*″(0) with the recently published article^30^ is included in Table 3, which proves that the present results are in excellent agreement. These results verify the accuracy of the present numerical code.

**Table 3:** The comparative values of *f*″(0) for several values of magnetic parameter $M$ when $S={E}_{c} =0$, for viscous fluid and hybrid nanofluid.

*M*	*φ*_1_ = *φ*_2_ = 0 (*Pr *= 1)		*φ*_1_ = 0.1, *φ*_2_ = 05 (*Pr *= 6.2)	
	Khashi'ie^30^	Present results	Khashi'ie^30^	Present results
	*f*″(0)	*f*″(0)	*f*″(0)	*f*″(0)
0	− 1.1737207	− 1.173720	− 1.251214	− 1.251215
0.5	− 1.3658145	− 1.365814	− 1.440257	− 1.440256
1	− 1.5357105	− 1.535710	− 1.608832	− 1.608835
1	− 1.8304897	− 1.830489	− 1.903452	− 1.903452
3	− 2.0848466	− 2.084846	− 2.159147	− 2.159148

Figures 1 and 2 are respectively plotted to measure the effects of Hartmann number $M$ with two different hybrid nano fluids i.e., $Cu{-}A{l}_{2}{O}_{3}$/${H}_{2}O$, $Cu{-}A{l}_{2}{O}_{3}$/${H}_{2}O{-}{C}_{2}{H}_{6}{O}_{2}$ and a simple nanofluid $A{l}_{2}{O}_{3}$/${H}_{2}O$ over the velocity profile ${f}^{^{\prime}}\left(\eta \right)$ and temperature profile $\theta (\eta )$. It is regarded that the values of ${f}^{^{\prime}}\left(\eta \right)$ and $\theta (\eta )$ are higher for the two hybrid nanofluids $Cu{-}A{l}_{2}{O}_{3}$/${H}_{2}O$, $Cu{-}A{l}_{2}{O}_{3}$/${H}_{2}O{-}{C}_{2}{H}_{6}{O}_{2}$ and is lower for the nanofluid $A{l}_{2}{O}_{3}/{H}_{2}O$. In other words, ${f}^{^{\prime}}\left(\eta \right)$ and $\theta (\eta )$ are more effective in the hybrid nanofluids. This is related to the fact that hybrid nanofluids possess multiples concentrations of nanoparticles and various densities of base fluids which make it possible to diminish the friction among the layers of fluid and consequently ${f}^{^{\prime}}\left(\eta \right)$ enhances for hybrid nanofluids with reference to a simple nanofluid. The addition of more nanoparticles increases the concentration of nanoparticles inside the base fluid which will increase the intermolecular collision. This enhances the kinetic energy which consequently enhances the temperature. It is also observed that ${f}^{^{\prime}}\left(\eta \right)$ declines whereas $\theta (\eta )$ boosts with increasing values of $M$. The existence of large number of nanoparticles in various base fluids improves the density of the associated hybrid nanofluids and thus the temperature of the coupled nanoparticles in the base fluids may be affected. Thus, hybrid nanofluids make superior performance in electronic components and other appliances.

The comparative influence of two different hybrid nano fluids i.e., $Cu{-}A{l}_{2}{O}_{3}$/${H}_{2}O$, $Cu{-}A{l}_{2}{O}_{3}$/${H}_{2}O{-}{C}_{2}{H}_{6}{O}_{2}$ and a simple nanofluid $A{l}_{2}{O}_{3}$/${H}_{2}O$ over the ${f}^{^{\prime}}\left(\eta \right)$ and $\theta (\eta )$ is found in Figs. 3 and 4. The influence of various values of suction $S$ is also shown in the two figures. It is argued that both ${f}^{^{\prime}}\left(\eta \right)$ and $\theta (\eta )$ are maximum for higher values of $S$. On the other hand, both ${f}^{^{\prime}}\left(\eta \right)$ and $\theta (\eta )$ are minimum for a simple nanofluid and is maximum for the two hybrid nanofluids i.e. by adding more nanoparticles ${f}^{^{\prime}}\left(\eta \right)$ and $\theta (\eta )$ rapidly enhances. Both these figures clarify that sooner the plots of ${f}^{^{\prime}}\left(\eta \right)$ and $\theta (\eta )$ boosts if we add more nanoparticles in the base fluids. Further, the two field of ${f}^{^{\prime}}\left(\eta \right)$ and $\theta (\eta )$ are also affected with the different rate of suction $S$, i.e. the plots of both ${f}^{^{\prime}}\left(\eta \right)$ and $\theta (\eta )$ boosts with the higher rate of suction $S$. Figures 5 and 6, respectively displays the effects of various aligned magnetic field angles $\beta$, the two hybrid nanofluids $Cu{-}A{l}_{2}{O}_{3}$/${H}_{2}O$, $Cu{-}A{l}_{2}{O}_{3}$/${H}_{2}O{-}{C}_{2}{H}_{6}{O}_{2}$ and a simple nanofluid $A{l}_{2}{O}_{3}$/${H}_{2}O$ over the ${f}^{^{\prime}}\left(\eta \right)$ and $\theta (\eta )$. It must be noticed that ${f}^{^{\prime}}\left(\eta \right)$ reduces with the increasing angle of inclination of aligned magnetic field $\beta$. Since the increment in the angle $\beta$ strengthens the magnetic flux and hence the Lorentz force increases. This opposite force causes the friction between the fluid layers which declines the fluid velocity ${f}^{^{\prime}}\left(\eta \right)$. On the other hand, $\theta (\eta )$ increases with increasing values of inclination angle $\beta$. One can see that both ${f}^{^{\prime}}\left(\eta \right)$ and $\theta (\eta )$ are dominant in the case of hybrid nanofluid as compared to the simple nanofluid. Figures 7 and 8, respectively represents the different effects of stretching $\gamma$ and the radiation $Rd$ over the temperature field $\theta (\eta )$. These effects are taken together with the consideration of two hybrid nanofluids $Cu{-}A{l}_{2}{O}_{3}$/${H}_{2}O$, $Cu{-}A{l}_{2}{O}_{3}$/${H}_{2}O-{C}_{2}{H}_{6}{O}_{2}$ and a simple nanofluid $A{l}_{2}{O}_{3}$/${H}_{2}O$. It is evident that $\theta (\eta )$ declines with the higher rate of stretching $\gamma$ as well as improves with the growing values of radiation parameter $Rd$. Since more heat is provided to the fluid with the higher values of $Rd$, which causes an increase in thermal boundary layer thickness and hence $\theta (\eta )$ increases.

The conduction of heat is well significant in manufacturing, electronic and digital apparatus. Hence it makes very important to use the flow of fluids in such type of accessories. The current research provides the different rates of heat transfer $Nu$ and friction drags ${C}_{f}$ in the flow of different hybrid nanofluids and a simple nanofluid composed of $Cu$, $A{l}_{2}{O}_{3}$ nanoparticles and ${H}_{2}O$, ${C}_{2}{H}_{6}{O}_{2}$ base fluids. The comparison of various values (curves) of ${C}_{f}$ and $Nu$ for the two hybrid nanofluids $Cu{-}A{l}_{2}{O}_{3}$/${H}_{2}O$, $Cu{-}A{l}_{2}{O}_{3}$/${H}_{2}O{-}{C}_{2}{H}_{6}{O}_{2}$ and a simple nanofluid $A{l}_{2}{O}_{3}$/${H}_{2}O$ along a stretching surface is respectively provided in Figs. 9 and 10. Additionally, the effects of Hartmann number $M$ is presented in the two figures, which shows that both ${C}_{f}$ and $Nu$ are the increasing functions of $M$. Further, it seems obvious that both ${C}_{f}$ and $Nu$ are dominant for the flow of simple nanofluid $A{l}_{2}{O}_{3}/{H}_{2}O$ and is least for the flow of hybrid nanofluids $Cu{-}A{l}_{2}{O}_{3}$/${H}_{2}O$, $Cu{-}A{l}_{2}{O}_{3}$/${H}_{2}O{-}{C}_{2}{H}_{6}{O}_{2}$. The existence of more nanoparticles makes it possible to diminish the friction among the layers of fluid which causes to down the resistance and consequently ${C}_{f}$ comes down for hybrid nanofluids with reference to a simple nanofluid. At the same time, Fig. 10 proves that the rate of $Nu$ is higher for the hybrid nanofluid and is lower for a simple nanofluid. Physically, the improvement in thermophysical properties of fluids occurs when base fluids contains more nanoparticles. Since, the addition of more nanoparticles increases the concentration of nanoparticles inside the base fluid which will increase the intermolecular collision. This enhances the kinetic energy which consequently enhances the temperature. Accordingly, the rate of heat transfer enhances for the hybrid nanofluids.

Figures 11 and 12 are plotted to see the effects of various values of suction $S$ over the ${C}_{f}$ and $Nu$, respectively. The influence of $S$ is considered under the effects of two different hybrid nanofluids $Cu{-}A{l}_{2}{O}_{3}$/${H}_{2}O$, $Cu{-}A{l}_{2}{O}_{3}$/${H}_{2}O{-}{C}_{2}{H}_{6}{O}_{2}$ and a simple nanofluid $A{l}_{2}{O}_{3}$/${H}_{2}O$ along a stretching surface. It is found that both ${C}_{f}$ and $Nu$ improves with the higher rate of suction however they act oppositely in case of stretching surface, i.e. higher rate of stretching declines ${C}_{f}$ and escalates $Nu$. Further, one can observe that the increasing values of aligned magnetic field angles $\beta$ rises the fraction drag ${C}_{f}$ whereas the rate of heat transfer $Nu$ comes down with the higher values of radiation $Rd$ as respectively seen in Figs. 13 and 14. This shows that the angle of inclination controls the intensity of magnetic field. Thus, the value of $\beta$ should be least if we need to reduce the friction drag on the surface. It is also evident that $Nu$ declines with the growing values of radiation parameter $Rd$ as shown in Fig. 14. Further, we noted that the different rates of stretching surface considerably effects the rate of heat transfer $Nu$ and skin friction coefficient ${C}_{f}$. One can finds that the higher values of $\gamma$ respectively provides the least values of ${C}_{f}$ and higher values of $Nu$, and vice versa. The rate of heat transfer and friction drag are the key properties of applied sciences and engineering entrust specially used in mechanical engineering process. On the basis of ${C}_{f}$ and $Nu$, it is possible to deduce that which fluid will be proficient in the heat transfer.

## Conclusion

This article allows us to provide the effect of several nanoparticles over the velocity, temperature, fraction drag, and heat transfer in the flow of different hybrid nanofluids. The evaluation covers the suspension of two distinct nanoparticles $Cu$ and $A{l}_{2}{O}_{3}$ in the combination of two different base fluids ${H}_{2}O$ and ${C}_{2}{H}_{6}{O}_{2}$. The conclusion drawn from this modern research is that the hybrid nanofluid ${Cu{-}A{l}_{2}{O}_{3} /H}_{2}O{-}{C}_{2}{H}_{6}{O}_{2}$ is quite effective in cooling and heating in comparison to the other hybrid nanofluid $Cu{-}A{l}_{2}{O}_{3}$/${H}_{2}O$, and a simple nanofluid $A{l}_{2}{O}_{3}/{H}_{2}O$. Based on these findings we could say that the suspension of multiple particles in the composition of two or more base fluids provides better rate of heat transfer, limits the friction drag and plays significant role in manufacturing, electronic and digital apparatus. Similarly ${f}^{^{\prime}}\left(\eta \right)$ and $\theta \left(\eta \right)$ are more effective in $Cu{-}A{l}_{2}{O}_{3}$/${H}_{2}O{-}{C}_{2}{H}_{6}{O}_{2}$ as compared to the $Cu{-}A{l}_{2}{O}_{3}$/${H}_{2}O$ and $A{l}_{2}{O}_{3}/{H}_{2}O$. Further, the value of inclination angle $\beta$ should be least if we need to reduce the friction drag on the surface. It is also evident that the rate of heat transfer $Nu$ declines with the growing values of radiation parameter $Rd$ as well as both skin friction ${C}_{f}$ and $Nu$ are the increasing functions of Hartmann number $M$.

## Abbreviations

*x*, *y*: Spatial coordinates [L] (*η*, *ξ*)
*f*(*y*): Normal component of the flow (*f*(η))
*ρ*_*nf*_: Density of nanofluids
*ρ*_*h*__*nf*_: Density of hybrid nanofluids
*ρ*_*f*_, *ρ*_*s*_: Density of base fluid and solid fraction (M/L^3^)
*ρ*_*f1*_, *ρ*_*s.*_: Density of *H*_2_*O* and *SiO*_2_ (M/L^3^)
*ρ*_*f2*_, *ρ*_*s2*_: Density of *C*_2_*H*_2_*F*_4_ and *Al*_2_*O*_3_ (M/L^3^)
*μ*_*n*__*f*_: Dynamic viscosity of nanofluid
*μ*_*h*__*n*__*f*_: Dynamic viscosity of hybrid nanofluid
*μ*_*f*_: Dynamic viscosity of base fluid (M/LT)
*μ*_*f1*_,* μ*_*f2*_: Dynamic viscosity of base fluids *H*_2_*O* and *C*_2_*H*_2_*F*_4_ (M/LT)
*v*_*n*__*f*_: Kinematic viscosity of nanofluid
*v*_*hn*__*f*_: Kinematic viscosity of hybrid nanofluid
*v*_*f*_, *v*_*s*_: Kinematic viscosity of base fluid and solid fraction (L^2^/T)
*k*_*n*__*f*_: Thermal conductivity of nanofluids
*k*_*hn*__*f*_: Thermal conductivity of hybrid nanofluids
*k*_*s*_, *k*_*f*_: Thermal conductivity of nanoparticles and base fluid (ML/T^3^K)
*k*_*s1*_, *k*_*f1*_: Thermal conductivity of nanoparticle *SiO*_2_ and base fluid *H*_2_*O*
*k*_*s2*_, *k*_*f2*_: Thermal conductivity of nanoparticle *Al*_2_*O*_3_ and base fluid *C*_2_*H*_2_*F*_4_
(*ρC*_*p*_)_*f*_: Heat capacity of nanofluids
(*ρC*_*p*_)_*hnf*_: Heat capacity of hybrid nanofluids
*Pr*: Prandtl number
(*ρC*_*p*_)_*s*_, (*ρC*_*p*_)_*f*_: Heat capacity of nanoparticles and base fluid (ML^2^/T^2^K)
(*ρC*_*p*_)_*s1*_, (*ρC*_*p*_)_*f1*_: Heat capacity of *SiO*_2_ and *H*_2_*O*
(*ρC*_*p*_)_*f2*_, (*ρC*_*p*_)_*f2*_: Heat capacity of *Al*_2_*O*_3_ and *C*_2_*H*_2_*F*_4_
*α*_*nf*_: Thermal diffusivity of nanofluids
*α*_*hnf*_: Thermal diffusivity of hybrid nanofluids
*α*_*s*_, *α*_*f*_: Thermal diffusivity of nanoparticle and base fluid (L^2^/T)
*C*_*f*_: Skin friction coefficient
*Nu*: Nusselt’s Number
*T*_*w*_, *T*_*∞*_: Reference and ambient temperature
*Re*_*x*_: Reynolds number
*γ*: Stretching parameter
*φ*: Nanoparticles concentration
*φ*_1_: Nanoparticle *SiO*_2_ concentration
*φ*_2_: Nanoparticle *Al*_2_*O*_3_ concentration
*G*: Stretching of surface
